# Supplementary figures and images for: MicroRNA‐transcriptome networks in whole blood and monocytes of women undergoing preterm labour
Source: J Cell Mol Med. 2019 Jul 24;23(10):6835–45. doi: 10.1111/jcmm.14567 (PMC6787570; doi:10.1111/jcmm.14567)

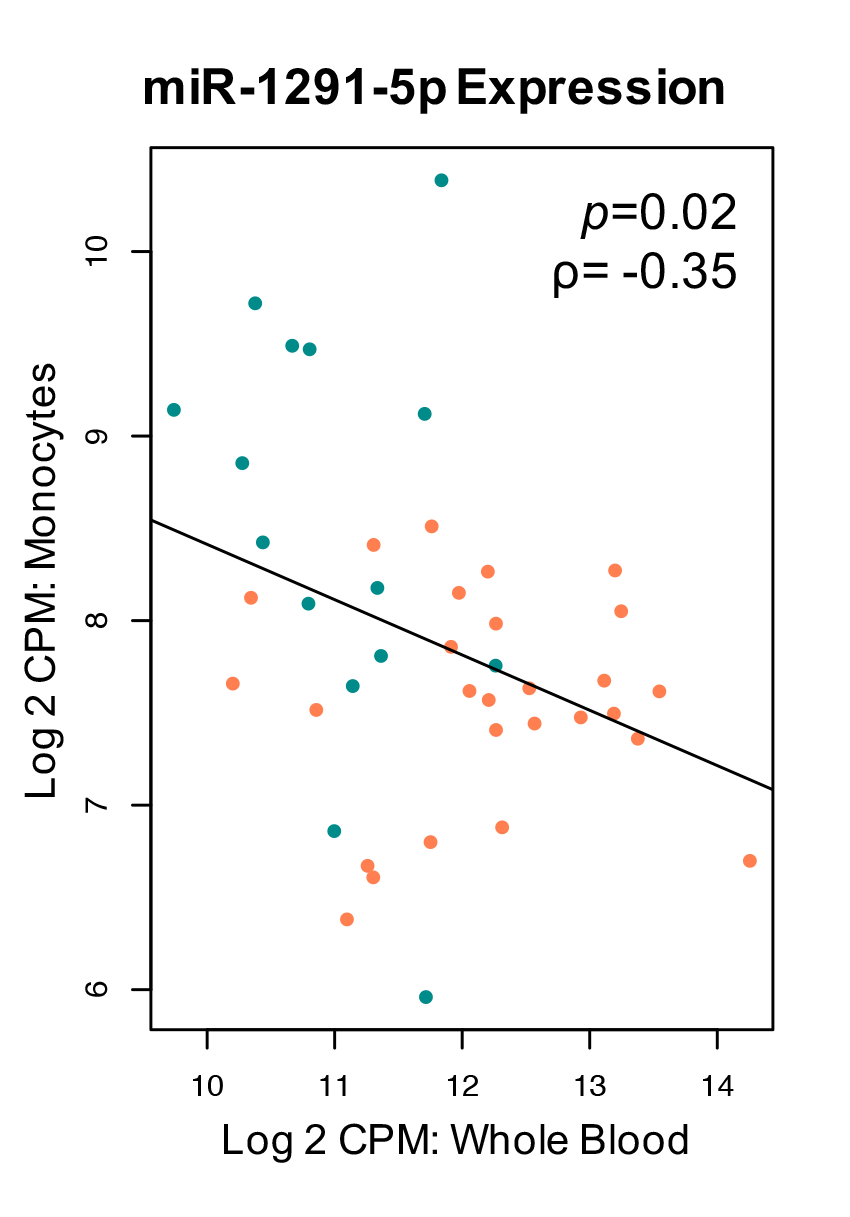

Supplement: Supplementary file 1 [file JCMM-23-6835-s001.tif]

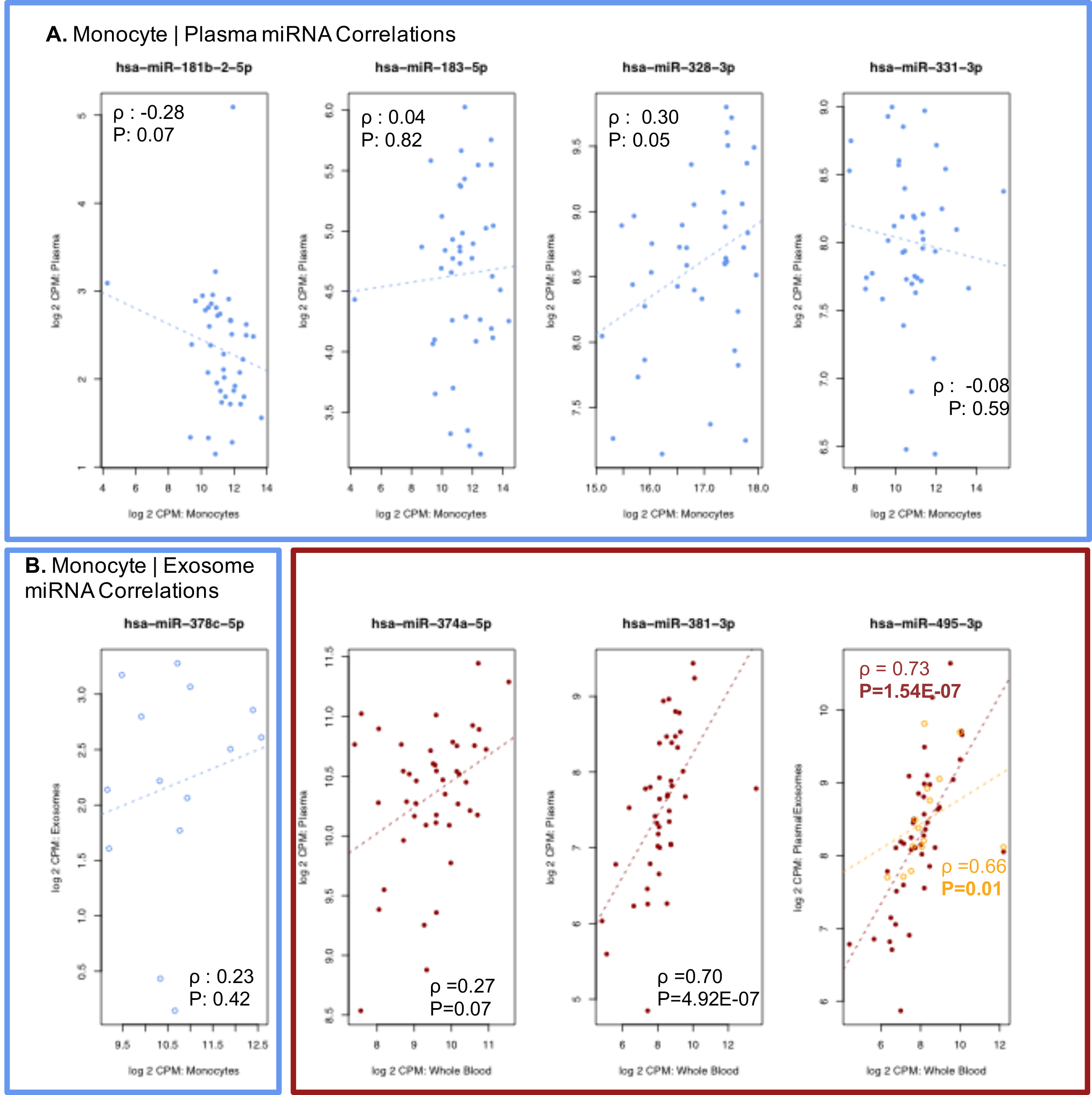

Supplement: Supplementary file 2 [file JCMM-23-6835-s002.tif]

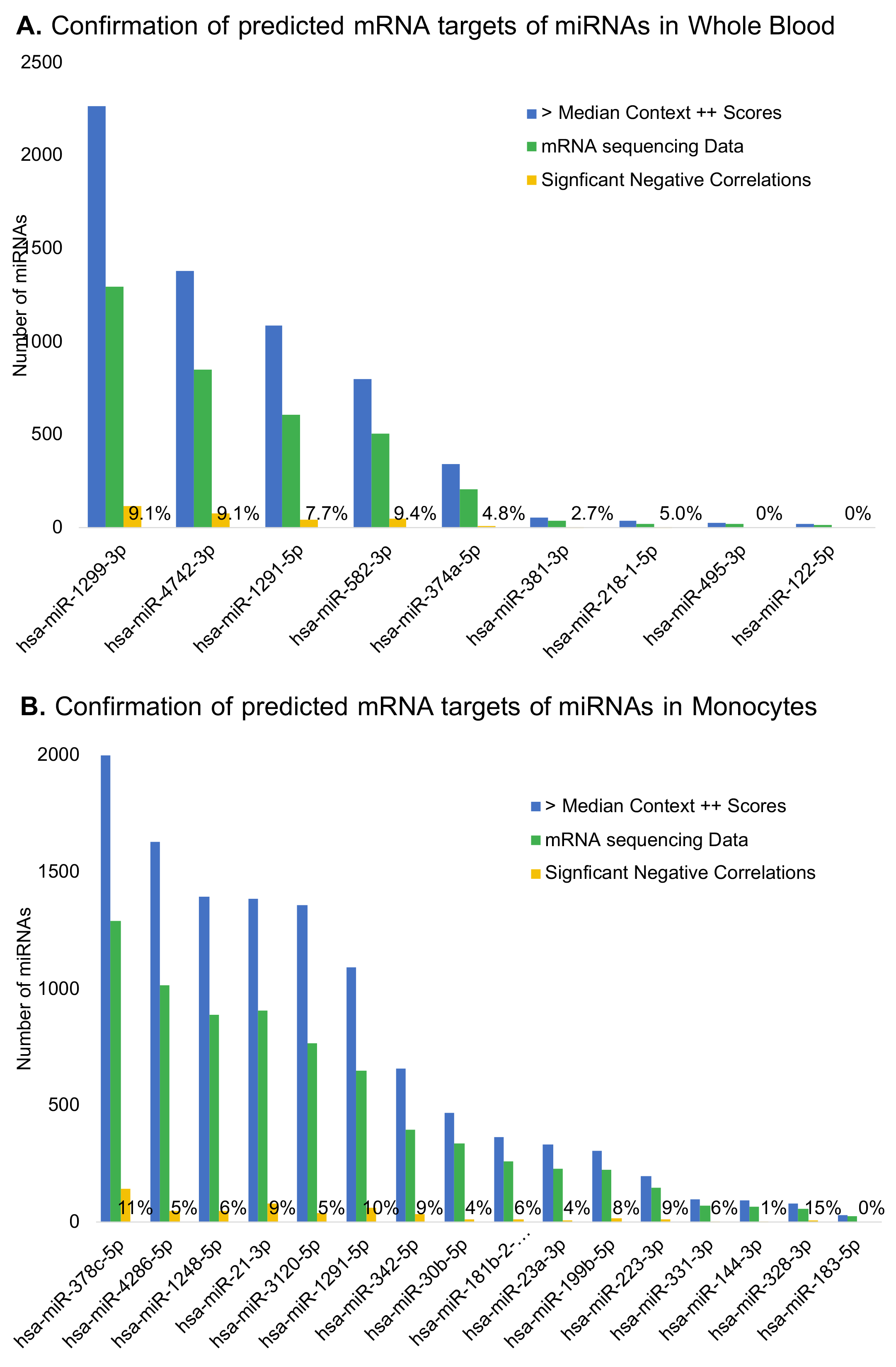

Supplement: Supplementary file 3 [file JCMM-23-6835-s003.tif]
